# Circulating annexin V positive microparticles in patients after successful cardiopulmonary resuscitation

**DOI:** 10.1186/cc10512

**Published:** 2011-10-25

**Authors:** Katrin Fink, Linda Feldbrügge, Meike Schwarz, Natascha Bourgeois, Thomas Helbing, Christoph Bode, Tilmann Schwab, Hans-Jörg Busch

**Affiliations:** 1 Department of Cardiology and Angiology, University Hospital of Freiburg, Hugstetter Str. 55, 79106 Freiburg im Breisgau, Germany

**Keywords:** Cardiopulmonary resuscitation, Circulating microparticles, Post-cardiac arrest syndrome, Ischemia/reperfusion syndrome, Endothelial apoptosis

## Abstract

**Introduction:**

Ischemia/reperfusion after cardiopulmonary resuscitation (CPR) induces systemic inflammatory response and activation of endothelium and coagulation, resulting in a post-cardiac arrest syndrome. We analysed circulating (annexin V+) microparticles and their conjugates in resuscitated patients.

**Methods:**

36 patients after successful resuscitation, 20 control patients with stable cardiac disease and 15 healthy subjects were included prospectively. Two blood samples were drawn, one immediately and one 24 hours after return of spontaneous circulation (ROSC) to detect (annexin V+) monocyte-derived microparticles (MMPs) or procoagulant (annexin V+) platelet-derived microparticles (PMPs) and conjugates of endothelial-derived (annexin V+) microparticles (EMPs) with monocytes (EMP-MC) or platelets (EMP-PC). Measurements were performed by flow cytometric analysis. Additionally, the effect of isolated microparticles on cultured endothelial cells was assessed by ELISA.

**Results:**

MMPs were significantly elevated immediately after ROSC compared to the cardiological control group (control; p < 0.01) and healthy subjects (healthy; p < 0.05) and persisted to be elevated in the following 24 hours after CPR (p < 0.05 vs. control and healthy, respectively). Procoagulant PMPs increased within the first 24 hours after ROSC (p < 0.01 vs. control and p < 0.005 vs. healthy). Conjugates of EMP with monocytes and platelets were both significantly elevated immediately after CPR (EMP-MC: p < 0.05 vs. control and p < 0.05 vs. healthy; EMP-PC: p < 0.05 vs. control and p < 0.05 vs. healthy), while only EMP-MC showed persisting high levels within 24 hours after CPR (p < 0.05 vs. control and p < 0.01 vs. healthy). MMP levels of ≥1.0/μL 24 hours after CPR predicted adverse outcome at 20 days (p < 0.05). Furthermore, isolated microparticles circulating in CPR patients early after ROSC led to enhanced endothelial apoptosis *ex vivo *compared to those of the healthy controls (p < 0.005).

**Conclusions:**

Resuscitated patients show substantially increased levels of different (annexin V+) microparticles and their conjugates immediately and 24 hours after cardiopulmonary resuscitation, suggesting an early onset of inflammation, an ongoing endothelial activation and a procoagulatory state. Additionally, microparticles of CPR patients may contribute to endothelial apoptosis. These results point to an involvement of microparticles in the development of the post-cardiac arrest syndrome.

## Introduction

Reperfusion following the return of spontaneous circulation (ROSC) after complete whole-body ischemia is an unnatural pathophysiological state created by successful cardiopulmonary resuscitation (CPR). Systemic ischemia/reperfusion response induces generalised tissue damage with a release of reactive oxygen species and endothelial-leukocyte interaction, resulting in a systemic inflammatory response [1,2], endothelial activation and injury [3-5], and coagulation abnormalities [6-8]. This so-called post-cardiac arrest syndrome shares many features with severe sepsis and may complicate the clinical course of resuscitated patients at the ICU [1,9].

Microparticles (MPs) are small vesicles, which typically range in size from 0.1 to 1.5 μm [9], shed from the plasma membrane into the extracellular space by most eukaryotic cells undergoing activation or apoptosis. They result from translocation of phosphatidylserine from the inner to the outer leaflet of the cell membrane where they express antigens characteristic of their cell of origin [10]. MPs are considered to act as diffusible messengers [11], to transport bioactive agents, and to initiate and mediate coagulation [12], inflammation and cell-cell interactions [13].

Monocyte-derived microparticles (MMPs) contain organized membrane receptors including ß2-integrins, like Mac-1 (CD11b/CD18). Therefore, they are capable of binding endothelial cells [11] and acting as competent inflammatory agonists by stimulation of cytokine release and up-regulation of endothelial adhesion molecules [14]. Additionally, MMPs play a crucial role in the initiation of endothelial dysfunction, endothelial thrombogenicity and apoptosis [14-16] and are capable of exposing a highly coagulant tissue factor [17]. Accordingly, elevated numbers of MMPs have so far been reported in patients with multiorgan failure, who developed severe disseminated intravascular coagulation [17,18], and in acute coronary syndromes, including acute myocardial infarction [19,20], underlining their inflammatory and procoagulant potential.

The second group, the platelet-derived microparticles (PMPs) are released by activated platelets [21] and are able to activate platelets and endothelial cells [22], monocyte adhesion [23] and the release of inflammatory cytokines [24] and induce endothelial apoptosis [25]. Consequently, elevated numbers of PMPs have been found in patients suffering from diseases associated with an increased risk of thromboembolic processes and vascular damage, including acute coronary syndromes [26], ischemic cerebrovascular disease [27] and peripheral arterial disease [28].

Endothelial-derived microparticles (EMPs) have been shown to be elevated after cardiopulmonary resuscitation [5] and in various states of disturbed endothelial function. EMPs have been shown to interact with monocytes or platelets to form circulating conjugates [29,30]. EMP-monocyte conjugates have been shown to be sensitive markers of disease activity in many disorders, including inflammatory and procoagulant conditions such as severe sepsis [31], multiple sclerosis [32], systemic lupus erythematosus, antiphospholipid syndrome [33] and venous thromboembolism [34]. An enumeration of EMP-platelet conjugates has been considered as a marker of activation of coagulation and endothelial cells as well as endothelial damage [30]. High numbers of these conjugates have been detected in the peripheral blood of patients with stable coronary disease [30].

### Intention of the study

In the present study, we aimed to investigate the effect of whole-body ischemia/reperfusion after successful CPR on the occurrence of different MPs and their conjugates in peripheral blood of resuscitated patients and their influence on patients' survival. Additionally the effect of MPs of resuscitated patients on endothelial cells was evaluated. As these MPs indicate and maintain inflammatory, coagulation and endothelial injuring processes, they are possible players in the field of post-cardiac arrest syndrome and may be indicators to predict outcome or possible targets of future therapeutic interventions.

## Materials and methods

### Study subjects

#### Patient recruitment

After the institutional approval of the Ethics Committee of our institution (EK-Freiburg 328/09), we included 36 patients who underwent CPR after cardiac arrest to measure PMPs and MMPs, EMP-monocyte and EMP-platelet conjugates. All patients were treated according to the international guidelines given by International Liaison Committee on Resuscitation in 2008 [35] and therefore received all therapeutic hypothermia. Results were compared with 20 patients with stable cardiac disease on a cardiological ward. As a second control, we performed measurements in 15 healthy subjects taking no medication and carrying no cardiovascular risk.

#### Inclusion and exclusion criteria

Inclusion criteria were CPR following cardiac arrest of any cause, in- or out-of-hospital with duration of CPR five minutes or longer. Resuscitation was performed according to the European Resuscitation Council Guidelines for Resuscitation 2005 [36-38]. Patients with malignant diseases were excluded from the study, because elevation of MPS has been reported to be increased in various malignancies [39]. Similarly, patients younger than 18 years, patients with sepsis or severe inflammation or trauma patients were excluded. Informed consent was obtained *post hoc *from patients surviving with good neurological outcome or from their relatives in case of the non-surviving. Informed consent was given by all patients in the control groups.

### Baseline characteristics

In resuscitated patients, data concerning the resuscitation period, for example, duration and location of CPR, time from cardiac arrest to start of mechanical resuscitation (no-flow time), presenting rhythm and presumable cause for cardiac arrest, were recorded. Baseline characteristics, such as age, gender, co-morbidities, cardiovascular risk profile, therapy received (medication, or coronary angiography), and some selected values of the local laboratory were collected. Additionally outcome parameters such as survival until hospital discharge, consecutive organ failure (definitions: acute kidney failure = increase in creatinine ≥0.3 mg/dL, or ≥1.5-fold climb in baseline creatinine within 48 hours; acute liver failure = increase in International Normalized Ratio ≥0.5 within 72 hours; acute heart failure = clinical signs of cardiac decompensation or cardiogenic shock), severity of disease (with the help of Sequential Organ Failure Assessment (SOFA)) and neurological outcome (on the basis of Glasgow Outcome Scale (GOS)) of survivors were recorded in both groups (Table 1).

**Table 1:** Baseline characteristics of resuscitated and control patients

		CPR		Control		*P *value
		*n *= 36		*n *= 20		
			**%**		**%**	
**Age (years)**		63.9 ± 2.1		65.7 ± 2.5		0.58/**ns**
**Gender**	Male	28	78	16	80	0.85/**ns**
	Female	8	22	4	20	
**Cause of CA or**	Cardiac	23	64	20	100	<0.005
**hospital admission**	Non-cardiac	10	28	0	0	
	Unknown	3	8	0	0	
**Coronary angiography**		23	64	12	60	0.78/**ns**
**Location of CPR**	out-of-hospital	27	75			
	in-hospital	9	25			
**Duration of CPR (min)**		21.8 ± 2.5				
**No-flow time (min)**		4.5 ± 0.8				
**Initial rhythm**	VT/VF	20	56			
	Asystole/PEA	16	44			
**Outcome**	Survival to discharge	14	39	20	100	
	GOS (survivors) at discharge	4.4 ± 0.2		5.0 ± 0.0		
	SOFA score day 1	11.0 ± 0.4				
	SOFA score day 2	10.6 ± 0.4				
**Medication at ICU**	Vasopressors	20	56	0	0	<0.001
	Thrombolysis	3	8	0	0	0.18/**ns**
	GPIIb/IIIa blockers	16	44	2	10	< 0.001
	Acetylsalicylic acid	25	69	15	75	0.66/**ns**
	Clopidogrel	21	58	12	60	0.90/**ns**
**Medication prior to event**	Statins	8	22	12	60	<0.01
**Consecutive organ**	Acute renal failure	8	22	0	0	<0.05
**failure**	Acute liver failure	3	8	0	0	0.54/**ns**
	Acute heart failure	4	11	0	0	0.29/**ns**
**Co-morbidities**	CAD	24	67	14	70	0.80/**ns**
	PAD	4	11	5	25	0.26/**ns**
	Chronic heart failure	2	6	3	15	0.34/**ns**
	Chronic renal insufficiency	4	11	2	10	0.99/**ns**
	Liver insufficiency	4	11	0	0	0.29/**ns**
	Pulmonary disease	9	25	6	30	0.69/**ns**
**Cardiovascular risk**	Hypertension	16	44	15	75	<0.05
**factors**	Hyperlipidemia	6	17	11	55	<0.005
	Diabetes	9	25	2	10	0.29/**ns**
	Smoking	7	19	8	40	0.10/**ns**
**Laboratory values**	Leukocytes/μL (day 1)	15.5 ± 1.1		7.7 ± 0.5		<0.001
	Leukocytes/μL (day 2)	13.7 ± 1.5		7.7 ± 1.0		<0.005
	Platelets/μL (day 1)	203.7 ± 12.3		231.9 ± 12.9		0.12/**ns**
	Platelets/μL (day 2)	183.9 ± 12.4		200.9 ± 41.0		0.7/**ns**
	CRP mg/L (day 1)	37.2 ± 11.3		8.7 ± 2.9		<0.05
	CRP mg/L (day 2)	56.8 ± 12.1		6.3 ± 1.4		<0.001

Basic data of the cardiopulmonary resuscitation and the cardiological control group (Control). Both groups are comparable in baseline variables including age, gender, incidence of coronary artery disease, coronary angiography, congestive heart failure and other relevant secondary disorders. There are slight but significant differences in distribution of cardiovascular risk factors as well as co-medication.

CA, cardiac arrest; CAD, coronary artery disease; CPR, cardiopulmonary resuscitation; CRP, C-reactive protein; GOS, Glasgow Outcome Scale; ns, statistically not significant; PAD, peripheral artery disease; PEA, pulseless electrical activity; SOFA, Sequential Organ Failure Assessment; VF, ventricular fibrillation; VT, ventricular tachycardia.

### Laboratory methods

#### Blood sampling and handling

Blood samples in resuscitated patients were collected immediately after admission to the ICU and 24 hours after ROSC, using an arterial line. In controls and healthy subjects, 20 mL blood was drawn from the arterial line in case of coronary catherization or by venipuncture on the second day and in healthy controls, respectively. Samples were drawn slowly, handled carefully and analysed immediately after sampling. For venipuncture, we used a 21-gauge butterfly needle and discarded the first 7.5 mL.

#### Antibodies

The following monoclonal antibodies (mAb) were purchased from Beckman Coulter (Marseille, France): anti-human CD61-FITC, anti-human CD14-FITC. Anti-human CD11b-FITC was purchased from Becton Dickinson (San Jose, CA, USA), annexin V and the mouse IgG_1_-PE and IgG_1_-FITC were from BD Biosciences Pharmingen (San Diego, CA, USA) and anti-CD62E-PE was obtained from Southern Biotech (Birmingham, AL, USA).

#### Flow cytometry analysis

Flow cytometry was performed using a three-colour FACSCalibur™ cytometer (BD Biosciences; San Jose, CA, USA) running Cell-Quest™ version 3.3 (BD Biosciences, San Jose, CA, USA), acquisition and analysis software. Instrument setup with individual settings for each antibody and calibration were performed with CaliBRITE™ beads (BD Biosciences, San Jose, CA, USA), according to the manufacturer's recommendations.

#### Sample preparation for measurement of MMPs, procoagulant PMPs, and EMP-platelet conjugates

Blood samples were collected in citrated tubes. According to the protocol of Hughes et al., samples were centrifuged for 10 minutes at 240 g at room temperature to obtain platelet-rich plasma and MPs were distinguished from platelets on the basis of their characteristic flow cytometric profile [40]. Supernatant was diluted 1:50 with Tyrode buffer and was incubated for 30 minutes at room temperature in the dark with fluorochrome-labelled antibodies. After the fixation of the tubes with 300 μL of CellFix™ (BD Biosciences, Erembodegem-Aalst, Belgium), samples were ready to be analysed by flow cytometry. MPs were counted on the basis of the events, positive for the cell-specific mAbs (see below) and values are given in counts/μL. Counts were calculated as events/36, because samples were analysed at a flow rate of 12 μL/min for 180 seconds (according to 36 μL/180 sec.).

#### Sample preparation for measurement of EMP-monocyte conjugates

For monocyte preparation, whole blood (100 μL) collected in citrated tubes was incubated with 2 mL FACS Lysing Solution™ (BD Biosciences, San Jose, CA, USA) at room temperature for 10 minutes. After centrifugation at 1,000 rpm for five minutes, supernatant was discarded, pellet washed twice with 2 mL PBS and resuspended in 50 μL PBS. Samples were incubated in the dark with anti-CD14-FITC and anti-CD62E. Mouse IgG_1_-PE or IgG_1_-FITC served as negative control. Following incubation, the samples were fixed with CellFix™ (BD Biosciences, Erembodegem-Aalst, Belgium) prior to analysis. Samples were analysed at a flow rate of 36 μL/min until 10,000 monocytes were collected in a specific monocyte gate. Values are given in counts/100 monocytes.

#### Identification of MMPs and procoagulant PMPs

**Figure 1:**
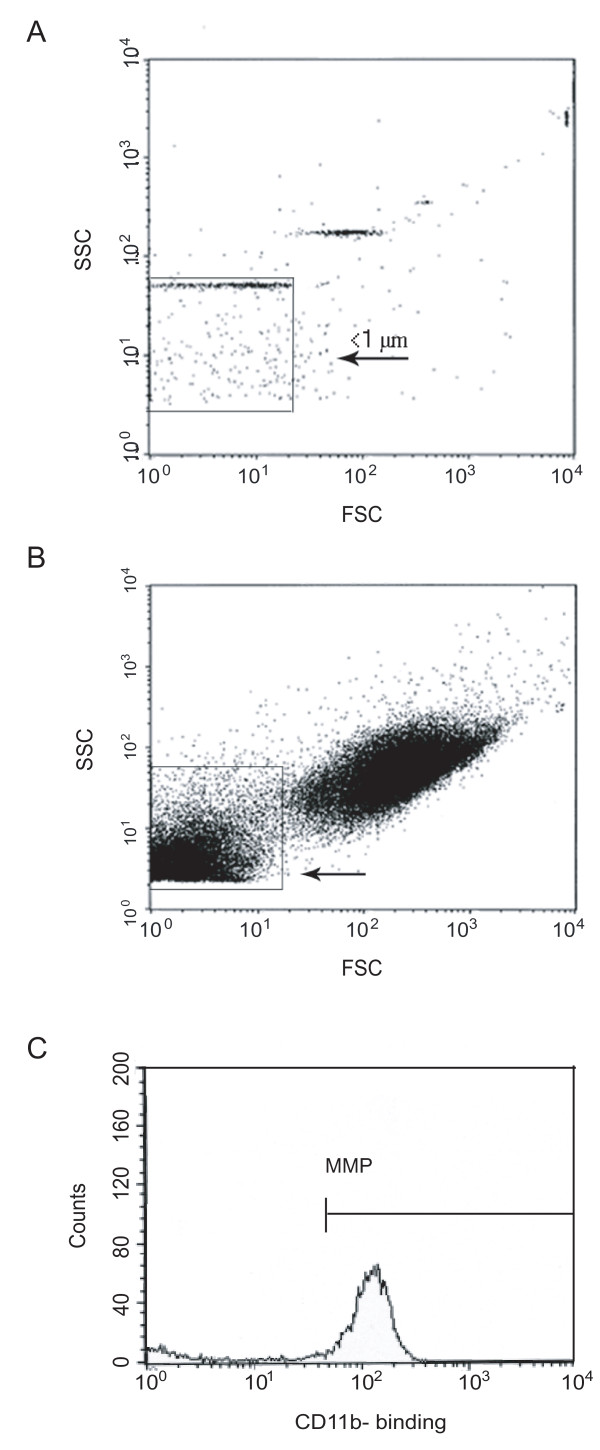
**Flow cytometric detection of microparticles in peripheral blood**. Three-colour flow cytometry evaluation of microparticles. **(a) **Detection of particles with a size of less than 1 μm by nano-beads, **(b) **then gating of microparticles in the lower left. **(c) **Events staining positive for the fluorochrome-labelled antibody directed against CD14 were identified as monocyte-derived microparticles (MMPs).

As CD11b is a subunit of Mac-1, representing an activated state of the monocytic MP subset, we chose anti-human CD11b-FITC for detection of MMPs. Procoagulant PMPs were identified by anti-human CD61-FITC, a beta-3-integrin on platelets. Mouse IgG_1_-PE or IgG_1_-FITC served as the negative control. During incubation, the cytometer was rinsed with FacsFlow™ (BD Biosciences, Erembodegem-Aalst, Belgium) for at least 30 minutes. MPs were gated by a size of less than 1 μm by using beads (Megamix™, Biocytex, Marseille, France) with a defined size of 0.9 μm (Figure 1). To check on the assumed identity as phosphatidylserine-expressing circulating MPs annexin V labelling was performed in the first measurements. Events were counted by triggering on the fluorescence signal above background noise.

#### Identification of EMP-monocyte and EMP-platelet conjugates

EMPs were identified by the activation-specific surface marker E-selectin (CD62E). Conjugates of EMPs with other cells were detected by employing strategic combinations of fluorescent-labelled antibodies depending on co-expression of endothelial marker CD62E with monocytes or platelets and expected bit-map location. Monocytes were identified and gated by positive staining for CD14, platelets by staining for CD61.

#### Measurement of IL-6 in resuscitated patients

In resuscitated patients, analysis was accompanied by the measurement of plasma IL-6 levels by chemiluminescent immunoassay in the central laboratory of the university hospital, Freiburg. Levels were compared with normal values of our institutional laboratory (<15 pg/mL).

#### Isolation of circulating MPs from resuscitated patients and healthy volunteers

MP isolation from citrated blood samples was performed according to the protocol of Boulanger et al. [41]. In brief platelet-free plasma was obtained by centrifugation at 11,000 g for two minutes and was subjected to further centrifugation at 13,000 g for 45 minutes. The MP pellets were resuspended in 100 μL of RPMI medium (containing Hepes and L-glutamine, from Lonza, Verviers, Belgium) and stored at -80°C until further analysis. Supernatant, containing the corresponding plasma of subjects were also kept and stored at -80°C to serve as the negative control. MP protein content was assessed by Bio-Rad Protein Assay (Bio-Rad Laboratories GmbH, München, Germany) and adjusted to the same values for each subgroup in the experiments.

#### Endothelial cell culture

Human umbilical vein endothelial cells (HUVEC) were obtained from Promocell™ (Heidelberg, Germany). The cells were cultured. For incubation with MPs, 10^4 ^cells per well were cultured in endothelial cell growth medium advanced (Provitro, Berlin, Germany) at 37°C in a 5% CO_2 _humidified atmosphere overnight. The following day cells were subjected to Optimem medium (Gibco, Invitrogen, Karlsruhe, Germany) and incubated with isolated MPs from resuscitated patients, healthy subjects or their corresponding plasma. MPs were added to equal protein content per well and incubated with HUVECs for 24 hours.

#### Ex vivo detection of apoptosis in cultured endothelial cells by ELISA

Detection and quantification of apoptosis in HUVECs after incubation with MPs or plasma of resuscitated and healthy probands was performed by the Cell Death Detection ELISA™ (Boehringer, Mannheim, Germany), according to the manufacturer's instructions. This is a colorimetric one-step sandwich ELISA for relative quantification of apoptosis by detection of histone-complexed DNA fragments (mono- and oligonucleosomes) after cell lysis.

### Statistical analysis

Continuous variables are given as means ± standard error of the mean. Data of CPR, control patients and healthy were compared by Mann-Whitney U- test, as appropriate. Comparisons between the different study populations and time points were performed using one-way repeated-measures analysis of variance, including an all-pair wise comparison. Correlations between selected variables were estimated by Spearman's rank correlation coefficient. Basic data and outcome were compared by Fisher's exact test, Chi-square test, and Student's t-test, respectively. Statistical significance was defined as a two-tailed *P *< 0.05. Analyses were performed with SPSS version 16.0 (SPSS Inc., Chicago, IL, USA).

## Results

### Baseline characteristics of patients

In the group of resuscitated patients, mean duration of mechanical resuscitation was 21.8 ± 2.5 minutes and estimated no-flow time was 4.5 ± 0.8 minutes. The presenting rhythm was ventricular fibrillation or ventricular tachycardia in 56% versus asystole or pulseless electrical activity in 44%. Twenty three patients (64%) presented a presumable cardiac cause for cardiac arrest and 14 patients (39%) survived until hospital discharge and mean GOS of survivors was 4.4 ± 0.2 at discharge. Severity of disease was evaluated by SOFA [42]. Mean SOFA score immediately after CPR was 11.0 ± 0.4 and 10.6 ± 0.4 24 hours later (Table 1). Average time from ROSC to first blood sampling was three hours and 42 minutes ± 48 minutes. The second blood sample was collected 25 hours and 48 minutes ± 42 minutes after ROSC.

Patients of the resuscitation and cardiological control group were comparable in baseline characteristics such as gender and age, as well as presence of a significant coronary artery disease (CAD) in medical history and proportion of subjects, which underwent coronary angiography during the blood sampling period of about 26 hours (Table 1). There were slight differences in cardiovascular risk profiles between the two groups with a higher incidence of hyperlipidemia in the control group. Resuscitated patients showed elevated leukocyte count and C-reactive protein levels but comparable platelet count compared with controls. Patients in the CPR group presented significantly higher incidence of acute renal failure, compared with control patients (Table 1).

All measurements were also performed in 15 healthy controls. Age at time of the study was 31.3 ± 1.9 years.

### MMPs and procoagulant PMPs

**Figure 2:**
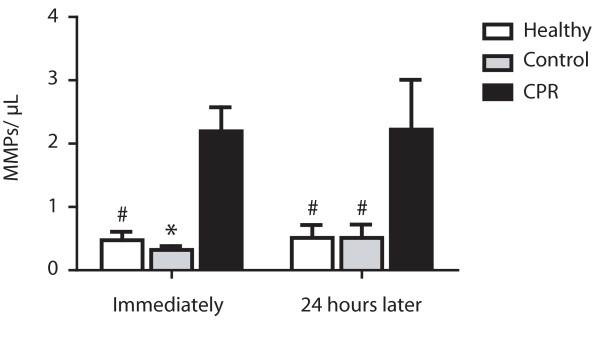
**Elevated monocyte-derived microparticles in patients after CPR**. Significant elevation of monocyte-derived microparticles (MMPs) in peripheral blood of resuscitated patients (cardiopulmonary resuscitation (CPR); black bars) immediately (left) and 24 hours after return of spontaneous circulation (right) compared with control patients with cardiac cause of hospital admission (Control; grey bars) and healthy subjects (Healthy; white bars). (* *P *< 0.01; ^# ^*P *< 0.05 versus CPR).

The mean number of MMPs in CPR patients was significantly increased immediately after ROSC compared with controls being hospitalized for a cardiac cause (mean number 2.2 ± 0.4 vs. 0.3 ± 0.06 events/μL; *P *< 0.01) or healthy controls (mean number 0.5 ± 0.1 events/μL; *P *< 0.05) and persisted in the 24 hours follow up (mean number vs. control: 2.2 ± 0.8 vs. 0.5 ± 0.2 events/μL; *P *< 0.05; or vs. healthy: 0.5 ± 0.2 events/μL; *P *< 0.05; Figure 2). In all groups, there was no significant change in MMP count in the 24 hour follow up (CPR: *P *= 0.22; control: *P *= 0.85; healthy: *P *= 0.48) and there was no correlation between monocyte count and MMP count detectable (r^2 ^= 0.01; *P *= 0.78).

**Figure 3:**
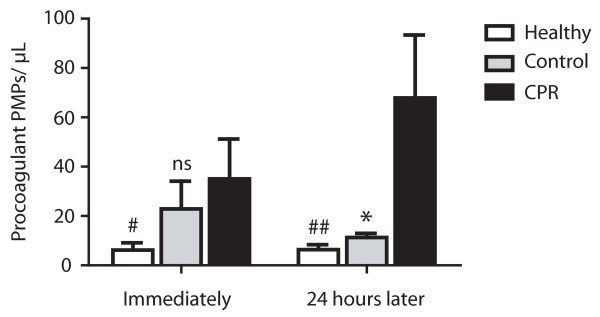
**Elevated procoagulant platelet-derived microparticles in patients after CPR**. Elevated number of procoagulant platelet-derived microparticles (PMPs) in resuscitated patients (cardiopulmonary resuscitation (CPR); black bars) compared with healthy controls (Healthy; white bars) immediately (left) and 24 hours after return of spontaneous circulation (right). Compared with control patients with cardiac cause of hospital admission (Control; grey bars), procoagulant PMP count was only slightly higher immediately after CPR (left), coming up to a significant elevation 24 hours later (right). (^## ^*P *< 0.005; * *P *< 0.01; ^# ^*P *< 0.05; ns, statistically not significant versus CPR).

Similarly procoagulant PMP levels were elevated in resuscitated patients. Whereas the mean number (35.1 ± 16.2 events/μL) was only significantly elevated compared with healthy subjects immediately after CPR (mean number 6.2 ± 2.9 events/μL; *P *< 0.05), there was only a slight but not significant increase detectable compared with the cardiological control group (mean number 22.8 ± 11.2 events/μL; *P *= 0.33). Twenty four hours after ROSC the mean number of procoagulant PMPs (67.9 ± 25.6 events/μL) in resuscitated patients was significantly higher than in the cardiological control group (mean number 11.3 ± 1.7 events/μL; *P *< 0.01) and in healthy subjects (mean number 6.4 ± 2.0 events/μL; *P *< 0.005; Figure 3). Increase in procoagulant PMP count in the first 24 hours in resuscitated patients failed to reach significance (*P *= 0.11) and there was also no significant difference between number of procoagulant PMPs in a follow up of 24 hours in both control groups (control: *P *= 0.74; healthy: *P *= 0.37). There was no correlation between platelet count and number of procoagulant PMP in all groups (r^2 ^= 0.02; *P *= 0.47).

### EMP-monocyte and EMP-platelet conjugates

**Figure 4:**
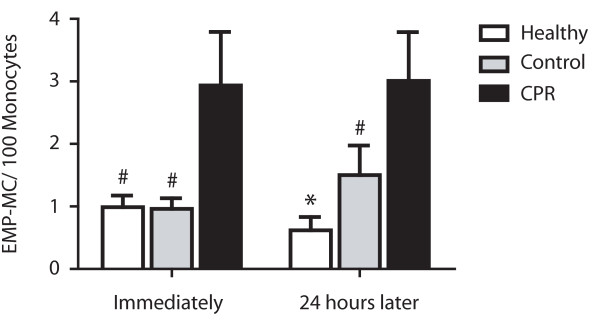
**Elevated conjugates of endothelial-derived microparticles and monocytes in patients after CPR**. Significant elevation of conjugates of endothelial-derived microparticles and monocytes (EMP-MC) in peripheral blood of resuscitated patients (cardiopulmonary resuscitation (CPR); black bars) immediately (left) and 24 hours after return of spontaneous circulation (right) compared with control patients with cardiac cause of hospital admission (Control; grey bars) and healthy subjects (Healthy; white bars). (* *P *< 0.01; ^# ^*P *< 0.05 versus CPR).

In resuscitated patients, significantly raised levels of EMP-monocytes conjugates were detectable compared with control patients and healthy controls immediately after ROSC (control: 2.9 ± 0.9 vs. 1.0 ± 0.2 events/100 monocytes; *P *< 0.05; healthy: 1.0 ± 0.2 events/100 monocytes; *P *< 0.05) and 24 hours after CPR (control: 3.0 ± 0.8 vs. 1.4 ± 0.5 events/100 monocytes; *P *< 0.05; healthy: vs. 0.6 ± 0.2 events/100 monocytes; *P *< 0.01; Figure 4). There was no significant difference in count of EMP-monocytes conjugates immediately after ROSC compared with the 24 hours follow up (CPR: *P *= 0.66; control: *P *= 0.95; healthy: *P *= 0.26) in all groups. There was no correlation between monocyte count and number of EMP-monocyte conjugates (r^2 ^= 0.07; *P *= 0.70).

**Figure 5:**
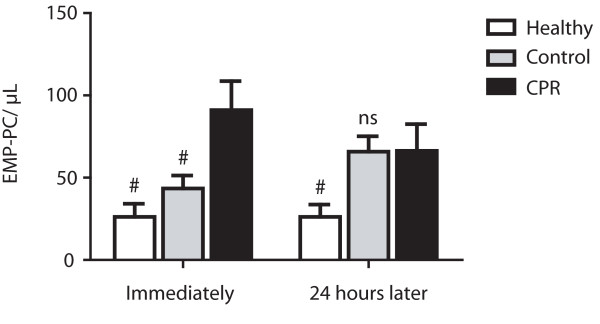
**Elevated conjugates of endothelial-derived microparticles and platelets in patients after CPR**. Significant elevation of conjugates of endothelial-derived microparticles and platelets (EMP-PC) in resuscitated patients (cardiopulmonary resuscitation (CPR); black bars) compared with control patients with cardiac cause of hospital admission (Control; grey bars) and healthy subjects (Healthy; white bars) immediately after return of spontaneous circulation (left). As values decline to levels comparable with the cardiac control group 24 hours after return of spontaneous circulation (right), there is a persistent significant elevation compared with healthy subjects. (^# ^*P *< 0.05; ns, statistically not significant versus CPR).

Number of EMP-platelet conjugates was significantly elevated immediately after ROSC (91.0 ± 17.7 events/μL) compared with patients in the cardiological control group (43.4 ± 7.8 events/μL; *P *< 0.05) and healthy controls (29.6 ± 8.0 events/μL; *P *< 0.05; Figure 5). Twenty four hours later, there was no more difference to the control group of patients being hospitalized for a cardiac cause (66.3 ± 17.3 events/μL vs. 65.8 ± 9.4 events/μL; *P *= 0.98), but an elevation compared with healthy subjects (26.2 ± 7.5 events/μL; *P *< 0.05) persisted. Decrease in numbers of EMP-platelet conjugates in the first 24 hours in resuscitated patients did not reach significance (*P *= 0.22) and there was also no significant difference between numbers of EMP-platelet conjugates in the control groups in a follow up of 24 hours (control: *P *= 0.06; healthy: *P *= 0.93). Comparable with EMP-monocyte conjugates, there was no correlation between platelet count and the number of EMP-platelet conjugates (r^2 ^= 0.01; *P *= 0.58).

### Prognostic value of circulating (annexin V+) MPs

To evaluate the prognostic value of different levels of (annexin V+) MPs, we compared the 20-day survival of groups divided by a certain cut-off value of the different (annexin V+) MPs measured immediately and 24 hours after ROSC.

**Figure 6:**
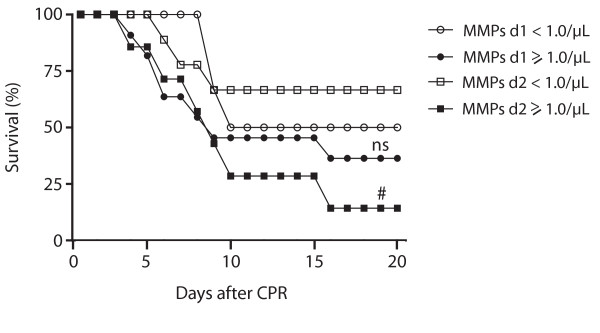
**20-day survival of patients with different levels of monocyte-derived microparticles after CPR**. Longer 20-day survival of resuscitated patients with levels of less than 1.0 monocyte-derived microparticles (MMPs)/μL (white quadrates) compared with patients with 1.0 MMPs/μL or more (black quadrates) 24 hours after return of spontaneous circulation (ROSC). There was no such difference detectable between the groups (white circles: <1.0 MMPs/μL; black circles ≥1.0 MMPs/μL) immediately after cardiopulmonary resuscitation (CPR). (^# ^*P *< 0.05; ns, statistically not significant versus <1.0 MMPs/μL; d1, day 1, immediately after ROSC; d2, day 2, 24 hours after ROSC).

In the MMP group, 50% of the patients with less than 1.0 MMPs/μL immediately after ROSC survived more than 20 days after CPR versus 36% of those with MMPs 1.0 MMPs/μL or more (*P *= 0.59), while 67% versus 14% survived longer than 20 days, when the MMP level was less than 1.0 MMPs/μL on the second day after CPR (vs. ≥1.0 μL; *P *< 0.05; Figure 6).

**Figure 7:**
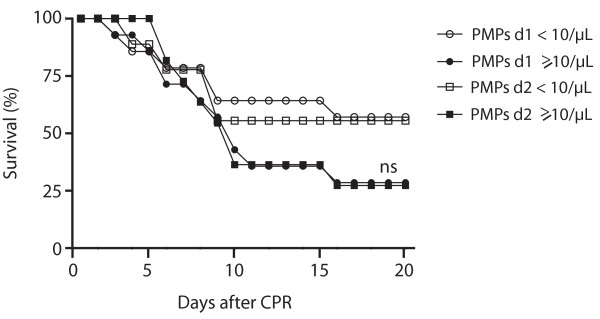
**20-day survival of patients with different levels of procoagulant platelet-derived microparticles after CPR**. Trend towards longer 20-day survival of resuscitated patients with levels of less than 10.0 procoagulant platelet-derived microparticles (PMPs)/μL (white circles: immediately after resuscitation; white squares: 24 hours later) compared with patients with 10.0 procoagulant PMPs/μL or more (black circles: immediately after resuscitation; black squares: 24 hours later) without statistic significant difference between the groups. (CPR, cardiopulmonary resuscitation; ns, statistically not significant versus <10.0 procoagulant PMPs/μL; d1, day 1, immediately after return of spontaneous circulation (ROSC); d2, day 2, 24 hours after ROSC).

Patients with procoagulant PMPs less than 10.0 PMPs/μL immediately after ROSC survived more than 20 days in 57 vs. 29% (*P *= 0.13) and in 56 vs. 27% (*P *= 0.20), when level of procoagulant PMP was 10.0/PMPs/μL or morein the 24 hour follow up (Figure 7).

There were no differences concerning the EMP conjugates with monocytes or platelets immediately after ROSC (42 vs. 43% for < or ≥ 5 EMP-monocyte conjugates/100 monocytes, respectively; *P *= 0.96 and 41 vs. 40% < or ≥ 50 EMP-platelet conjugates/μL, respectively; *P *= 0.95). Twenty four hours later, there was still no significant difference between patients presenting with less than five and five or more EMP-monocyte conjugates/100 monocytes (50 vs. 40%; *P *= 0.67) or between subjects with less than 50 related to those with 50 or more EMP-platelet conjugates/μL (31 vs. 50%; *P *= 0.38).

### IL-6 levels in resuscitated patients

Resuscitated patients showed significantly elevated plasma levels of IL-6 immediately (430.5 ± 148.6 pg/mL; *P *< 0.05) and 24 hours after ROSC (1067.5 ± 185 pg/mL; *P *< 0.01) compared with normal values of our institutional laboratory (<15 pg/mL).

### MP-induced apoptosis in HUVECs

**Figure 8:**
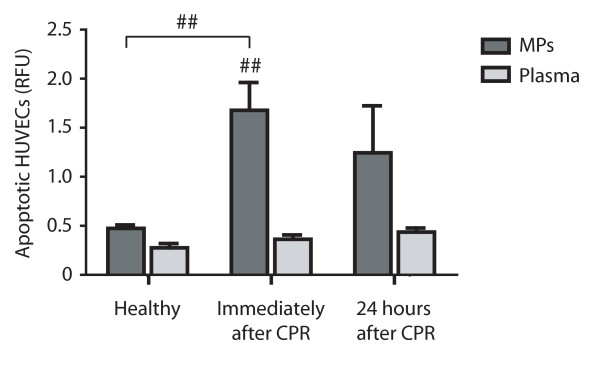
**Enhanced apoptosis after stimulation of endothelial cells with microparticles of resuscitated patients**. Enhanced apoptosis of endothelial cells (HUVECs) after stimulation with microparticles (MPs) of resuscitated patients (dark grey bars) compared with their plasma (light grey bars) and compared with endothelium stimulated with MPs of healthy controls (Healthy; left). This apoptotic effect is significant for MPs isolated from patients early after resuscitation (Immediately after cardiopulmonary resuscitation (CPR); middle), but not for MPs isolated from resuscitated patients in a 24-hour course after cardiac arrest (24 hours after CPR; right). (^## ^*P *< 0.005 versus CPR or versus plasma, respectively).

Endothelial cells viability was evaluated *ex vivo *by a DNA fragmentation ELISA after incubation with MPs or plasma obtained from resuscitated patients and healthy subjects. MPs of resuscitated patients isolated immediately after CPR resulted in significantly enhanced endothelial apoptosis compared with plasma of CPR patients (1.7 ± 0.3 vs. 0.4 ± 0.05 RFU; *P *< 0.005), as well as compared with purified MPs of healthy controls (1.7 ± 0.3 vs. 0.5 ± 0.04 RFU; *P *< 0.005). This elevated apoptosis rate was notable only in trend for MPs isolated 24 hours after ROSC, but there were no more statistically significant differences, neither in comparison to plasma (1.2 ± 0.5 vs. 0.4 ± 0.04 RFU; *P *= 0.11) nor to isolated microparticles of healthy (1.2 ± 0.5 vs. 0.5 ± 0.04 RFU; *P *= 0.15; Figure 8).

## Discussion

Our study demonstrates that different subtypes of (annexin V+) MPs and their conjugates are elevated and may influence the clinical course of patients after successful CPR. The main findings were a marked elevation of MMPs and EMP-monocyte conjugates immediately and in the following 24 hours after ROSC compared with control patients hospitalized for cardiac causes and healthy subjects. Additionally, we found increasing levels of procoagulant PMPs in the 24 hour follow up after CPR and a significant increase of EMP-platelet conjugates early after ROSC compared with both control groups. These results suggest an early onset of inflammation, an ongoing process of endothelial activation and a procoagulatory state and thereby, an involvement of (annexin V+) MPs in the development of the post-cardiac arrest syndrome. Additionally, we found MMP levels of 1.0 MMPs/μL or more on the second day after ROSC to be a prognostic value to predict outcome at 20 days after cardiac arrest.

As MPs are known to be elevated in CAD patients [20,30], we compared resuscitated subjects with patients with stable cardiac disease, mostly presenting CAD and undergoing coronary catherization in a similar proportion, to exclude possible effects caused by CAD or coronary intervention in the resuscitation group. Additionally, we included a smaller population of healthy volunteers.

The most impressive finding of the study is the strong elevation of MMPs and EMP-monocyte conjugates after CPR. The considerable increase immediately after ROSC was followed by persistently high levels in the first 24 hours after ROSC. Formation of leukocyte-derived MPs in general is enhanced by inflammatory stimuli and MMPs are known to be important *in vivo *markers of neutrophil activation [43]. They are capable of expression of selectins and integrins, which makes them candidates for playing a crucial role in inflammation and cell signalling [44], suggesting a distinct inflammatory process occurring in patients after CPR. Additionally, MMPs are competent inflammatory mediators to induce endothelial activation and cytokine production [14], thus potentially contributing to endothelial activation and dysregulation [5,15,45]. On the other hand, EMPs, shed by the activated endothelium, have been shown to be elevated after CPR [5] and are known to bind to monocytes in a time- and concentration-dependant manner [29] to form EMP-monocyte conjugates. Once bound to monocytes, they activate them to enhanced expression of integrins and migration through the endothelial cell layer [29,32]. Our results are in line with various reports of an inflammatory in patients after CPR [1-4]. Accordingly, IL-6 levels were also significantly elevated in resuscitated patients at both time points, whereas the preserved elevation of MMPs and EMP-monocyte conjugates in our study provides evidence for an ongoing inflammatory state in the first 24 hours of reperfusion. This is also reflected by a persistent elevation of inflammatory markers such as leukocyte count and C-reactive protein immediately and 24 hours after CPR compared with control patients. We could not observe any correlation between monocyte count and number of MMPs, or EMP-monocyte conjugates so that the results give the impression that (annexin V+) MP and conjugate counts are independent markers of inflammation and not only elevated arising from a general elevation of leukocytes or monocytes in resuscitated patients.

Other players in this field of activated coagulation and leukocyte/endothelial interaction are PMPs. As the phospholipid-dependent procoagulant activity of PMPs is limited to the annexin V positive subpopulation [46], we measured procoagulant PMPs in this study, which were also elevated after CPR. PMPs in general are formed by the attachment of platelets to the vascular wall. Besides their procoagulant properties they are able to directly activate platelets [22]. Furthermore, they are able to increase the adherence of monocytes to endothelial cells by the up-regulation of adhesion molecules [23]. Therefore, Jy et al. suggested a possible role of PMPs as an activator of neutrophils in ischemic injury, thrombosis and inflammation [47]. Our data match studies which revealed an increase of procoagulant PMPs in patients with ischemia/reperfusion, vascular injury and a risk of thromboembolism such as acute coronary syndromes [20,26] or ischemic stroke [27]. As levels of procoagulant PMP were elevated compared with controls presenting CAD in a similar proportion to the resuscitated, we can exclude the possibility that CAD alone contributed to this effect.

Procoagulant PMPs were significantly elevated immediately after CPR compared with healthy subjects, but failed to show a significant difference with respect to the cardiac patients. This could be due to application of GPIIb/IIIa receptor antagonists during coronary intervention in a substantial amount of patients (42 vs. 10% in controls) [20]. In point of fact, the subgroup of patients receiving GPIIb/IIIa receptor antagonists had a trend towards lower counts of procoagulant PMP, but there was no statistical difference between the two subgroups (data not shown). Another possible explanation is given by Nomura et al., who could not detect elevated levels of free PMPs in patients with multiple sclerosis and supposed that this was due to an absorbance of most of the free PMPs to leukocytes, contributing to the increased platelet-leukocyte conjugates in their study [24]. Finally, high shear stress can initiate both platelet aggregation and shedding of procoagulant-containing MPs [48] and the observed elevation could therefore also possibly be due to shear stress occurring during mechanical resuscitation.

Finally, our study revealed higher EMP-platelet conjugates immediately after CPR. These conjugates originate from binding of EMPs to activated platelets and have so far only been described by Héloire et al. in patients with CAD. The authors hypothesise that these conjugates originate from strongly activated and damaged endothelial cells and activated platelets and are able to contribute to thrombus formation by undergoing sequestration in peripheral vessels [30]. In synopsis with our previous findings of a severe endothelial damage early after resuscitation [5], the elevation of EMP-platelet conjugates in resuscitated patients could reflect endothelial injury and microcirculatory disorders occurring after CPR.

Interestingly, patients presenting with less than 1.0 MMP/μL on the second day after CPR showed a better 20-day survival after CPR compared with those with 1.0 or more MMPs/μL. Accordingly, we found this in trend too for patients with less than 10.0 procoagulant PMPs/μL immediately and 24 hours after CPR, although there was no statistical significant difference. Therefore, we suggest a potential prognostic value of MMP counts on the second day after CPR. It seems likely that the small number of subjects in each subgroup is due to the fact that these notable differences did not reach statistically significant differences.

Additionally, we provide evidence for enhanced apoptosis of endothelial cells under the influence of MPs of resuscitated patients *ex vivo*. This is in line with other publications reporting of endothelial apoptosis under the influence of different MP subpopulations, such as *in vitro *generated MMPs [16] or EMPs [49]. Additionally Gambim et al. showed that endothelial cells exposed to PMPs isolated from patients with septic shock undergo apoptosis [25]. In comparing the effect of MPs with the effect of plasma of the same patients, we can exclude an apoptotic effect of certain plasma compounds occurring after CPR. As we tested the whole amount of circulating MPs in CPR patients we cannot identify a certain subpopulation contributing to or even initiating apoptosis in our study.

A limitation of the study could be the sample size, which is relatively small (*n *= 36) with a considerable heterogeneity of the patients, particularly in the CPR group. Although statistical analysis reveals clear differences between the groups, these results should be validated in a larger study.

## Conclusions

With this study, we can demonstrate that different (annexin V+) MPs and their conjugates are elevated in patients suffering from cardiac arrest and CPR and might reflect pathophysiological processes, including inflammation, activation of coagulation and endothelial injury, following whole-body ischemia/reperfusion. We provide evidence for induction of endothelial apoptosis by MPs circulating in patients after CPR and we can show a prognostic value in predicting 20-day survival after resuscitation by MMP levels on the second day after CPR. Therefore, we suggest an involvement of MPs in the development of the post-cardiac arrest syndrome.

## Key messages

• Patients after successful CPR show a significant activation of inflammation.

• Platelet activation seems to occur 24 hours after CPR.

• Elevated conjugates of EMPs with monocytes or platelets point to endothelial and platelet activation and activation of inflammation.

• MPs of resuscitated patients induce endothelial apoptosis.

• MMP levels 24 hours after ROSC seem to have prognostic value.

## Abbreviations

CAD: coronary artery disease; CPR: cardiopulmonary resuscitation; ELISA: enzyme-linked immunosorbent assay; EMP: endothelial-derived (annexin V+) microparticles; GOS: Glasgow Outcome Scale; HUVEC: human umbilical vein endothelial cells; IL-6: interleukin-6; mAb: monoclonal antibodies; MMP: monocyte-derived (annexin V+) microparticles; MP: microparticles; PBS: phosphate buffered saline; PMP: platelet-derived (annexin V+) microparticles; ROSC: return to spontaneous circulation; SOFA: Sequential Organ Failure Assessment.

## Competing interests

The authors declare that they have no competing interests.

## Authors' contributions

KF was responsible for the conception and the design of the study, for the acquisition, analysis, and interpretation of data as well as for the writing of the manuscript. LF recruited patients, drew blood samples and acquired data for MP measurements. MS was involved in conception and the design of the study, as well as analysis and interpretation of data and helped in trouble-shooting. NB acquired data and helped in patients' recruitment. TH revised the manuscript critically and gave important advices for completion and in the major revision process. CB contributed important intellectual content and gave final approval for the version to be published. TS contributed to the writing of the manuscript and was responsible for revising it critically. HJB participated substantially in the conception of the study, analysis, and interpretation of data as well as in the writing of the manuscript. All authors read and approved the final manuscript for publication.
